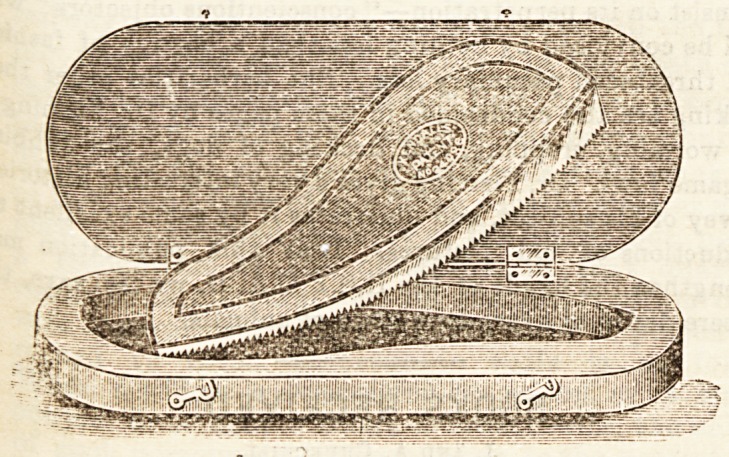# New Appliances and Things Medical

**Published:** 1898-11-12

**Authors:** 


### NEW APPLIANCES AND THINGS MEDICAL.

[We shall be glad to receive, at our Office, 28 & 29, Southampton Street, Strand, London, W.C., from the manufacturers, specimens of all new preparations and appliances which may be brought out from time to time.]

### KENDALL'S PATENT SAW FOR REMOVING PLASTER OF PARIS AND OTHER BANDAGES.

(E. G. Kendall, 53, High Street, Harborne, Birmingham.)

This is a most useful and convenient little instrument. Everyone knows the difficulty which is sometimes experienced in removing a starch bandage or a plaster of Paris splint. If the plaster is at all thick nothing but a saw will satisfactorily divide it, while the ordinary saw is a most dangerous instrument for the purpose. With this saw, however, all risk of injuring the skin is obviated. The edge of the saw forms a segment of a circle, and the blade is fixed eccentrically between two pieces of wood in such a manner that the cutting edge projects more at the one end than at the other. When being used the wood forms a guard which limits the depth to which the saw can cut, and this depth varies according to the portion of the curved edge of the saw which is brought into use. If the bandage is thin, and a shallow cut is required, one end of the saw alone is used; if a deep cut is wanted, the other end; while intermediate portions give intermediate depths. The instrument is extremely simple and its utility is unquestionable. The price in case is 12s. 9d., post free.

### "WAWKPHAR."

(The Wawkphar Co., 39, Victoria Street, S.W.)

"Wawkphar" is an antiseptic powder for the use of persons "troubled with tender feet, or liable to chafing, overheating, or excessive perspiration." It is said to be recommended by the War Office for use in the army "for strengthening and soothing the feet on marches, or any work demanding long standing or severe strains upon the limbs." Examination of the powder showed it to consist of ingredients possessing antiseptic, absorbent, and emollient properties which would be most efficacious for the purposes indicated. It is perfectly free from metallic substances of a poisonous nature.

### HELLER'S HARDWARE SHELF BOX.

(James Keeves and Sons, Boundary Street, Shoreditch, London, N.E.)

One's first question naturally is, What is a shelf box? Well, it is a box, or rather a number of boxes, made to stand on shelves with the intent that, instead of having odds and ends piled up loose, they may lie neatly in boxes as if they were in drawers. Primarily, the contrivance is intended for chemists and druggists and people who have shops, and the idea is that they can place such goods as are constantly in requisition in these shelf boxes; but it seems to us that there are many things in many surgeries which do not fit very well into any drawer that is available, and so lie about greatly to the detriment of neatness, which would quite easily go into these shelf boxes, for they can be had in all sorts of sizes to fit any shelves that may be handy.

## Figures and Tables

**Figure f1:**